# Laparoscopic hepatopancreaticoduodenectomy for synchronous gallbladder cancer and extrahepatic cholangiocarcinoma: a case report

**DOI:** 10.1186/s12957-022-02628-9

**Published:** 2022-06-09

**Authors:** Guo-Liang Yao

**Affiliations:** Department of General Surgery, The First Affiliated Hospital of Henan University of Science and Technology, 24 Jinghua road, Luoyang, 471000 China

**Keywords:** Hepatopancreaticoduodenectom, Hepatopancreatoduodenectomy laparoscopy, Gallbladder cancer, Extrahepatic cholangiocarcinoma

## Abstract

**Background:**

Hepatopancreaticoduodenectomy (HPD) is one of the most complex procedures, and it is very rarely reported. Laparoscopic HPD (LHPD) is even rarer. To date, there are only 3 reports of LHPD for locally advanced gallbladder cancer (GBC) or extrahepatic cholangiocarcinoma (ECC). This is the first report of LHPD for synchronous GBC and ECC.

**Case presentation:**

A 75-year-old female patient complained of jaundice for 2 weeks without fever or abdominal pain. She was diagnosed with synchronous GBC and ECC. After a comprehensive preparation, she underwent a laparoscopic pancreaticoduodenectomy and resection of hepatic segments of IVb and V, and her digestive tract reconstruction followed Child’s methods. She was discharged on the 12th day postoperatively without pancreatic leakage, biliary leakage, or liver failure.

**Conclusions:**

LHPD is safe and feasible for selected cases of GBCs or ECCs.

## Background

Hepatopancreaticoduodenectomy (HPD) is one of the most complicated operations which includes hepatectomy and pancreaticoduodenectomy. This operation was first reported in 1974 by Kasumi for locally advanced gallbladder cancer (GBC) [1]. Almost half a century passed, this procedure was not universally accepted. In total, no more than 1000 HPDs have been reported [2, 3] for the past 50 years because of the high morbidity and mortality [4–6]. After a comprehensive search of PubMed using the terms of laparoscopy, hepatopancreaticoduodenectomy, hepatopancreatoduodenectomy, hepatopancreatectomy, pancreaticoduodenectomy, and hepatectomy, there were only 3 reports [7–9] involving 3 laparoscopic HPDs (LHPD) for locally advanced GBC or extrahepatic cholangiocarcinoma (ECC) which were list in detail in Table 1. To our knowledge, this case is the first LHPD for concurrent GBC and ECC.

**Table 1:** Summary of the published cases

Author	Country	Year	Diagnosis	Gender	Age (y)	Operation	Operation duration (min)	Intraoperation blood loss (ml)	Hospitalization (d)	Main complication
Zhang MZ	China	2014	ECC	Male	61	LPD + LRH	600	450	16	Biliary leakage
Chong EH	South Korea	2019	ECC	Female	73	LPD + LLH^a^	510	350	16	Cystitis
James M	India	2021	GBC	Male	73	LPD + segments of IVb + V	610	500	12	Delayed gastric empty

*LPD* Laparoscopic Pancreaticoduodenectomy, *LRH* Laparoscopic Right Hemihepatectomy, *GBC* Gallbladder Cancer, *LLH* Laparoscopic Left Hemihepatectomy

^a^Laparoscopic resection and robotic reconstruction

## Case report

**Fig. 1:**
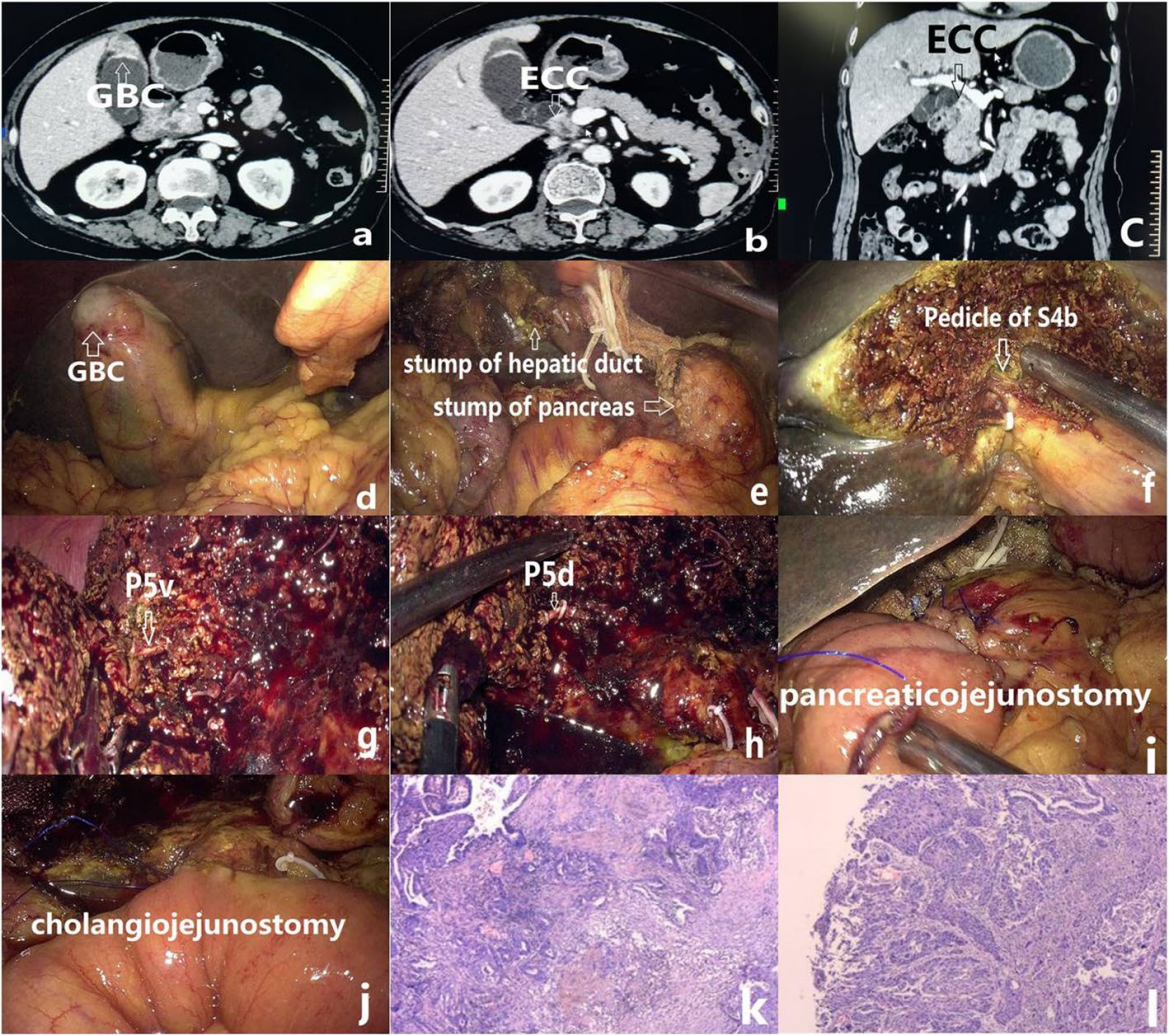
**a** Thickened wall of gallbladder by enhanced CT. **b** Thickened wall of common bile duct by enhanced CT. **c** Thickened wall of common bile duct by enhanced CT. **d** Suspected gallbladder cancer during operation. **e** Stump of the pancreas and hepatic duct. **f** Hepatic pedicle of segment IVb. **g** Ventral portal vein of segment V. **h** Dorsal portal vein of segment V. **i** Pancreaticojejunostomy. **j** Cholangiojejunostomy. **k** Pathology of gallbladder (× 100). l pathology of a common bile duct (× 100)

A 75-year-old female patient complained of jaundice without fever or abdominal pain for 2 weeks and was diagnosed with ECC with GBC by enhanced CT and MRI. She denied any history of cardiovascular or pulmonary system problems. Her physical examination was negative except for jaundice of her skin and sclera. Her routine laboratory tests were as follows: hemoglobin 108 g/L, total bilirubin 222.8 μmol/L, direct bilirubin 158 μmol/L, plasma albumin 35.2 g/L, ALT 297 U/L, AST 192 U/L, ALP 403 U/L, and γGGT 394 U/L. Her tumor markers were normal, except for a mildly elevated Ca19-9 of 34.06 U/ml. Her hemostatic function was normal. An enhanced CT and MRI revealed that there was irregular thickening of the common bile duct wall and the gallbladder (Fig. 1). Cytological examination by ERCP was performed with the result of a malignant tumor of the lower common bile duct.

The patient was placed in a supine position with the legs split. Five ports were employed for the operation with an additional port located subxiphoid for splitting the liver. First, resection of the gallbladder was planned and then fast-frozen pathology was performed due to the presence of a malignant tumor. Pancreaticoduodenectomy was performed by artery-first approach. First, the superior mesenteric artery was retropancreatically dissected, the stomach and pancreatic neck were transected, and finally, the common hepatic duct was transected. The hepatectomy was performed along the intrahepatic Glissonean pedicle. The ligamentum teres hepatis was dissected to reveal the pedicle of a segment of IVb. The pedicle was transected, and the liver was continuously split until reaching the right anterior branch of the portal vein. Split liver and transected middle hepatic vein to further reveal the pedicle of segment V and then transected it (Fig. 1).

Reconstruction was performed following Child’s methods. Pancreaticojejunostomy was completed by the modified Blumgart method. Cholangiojejunostomy was completed by 4–0 polydioxanone in a manner of a continuous suture. The gastroenterostomy was completed by an endolinear stapler.

The operation lasted 380 min with an estimated blood loss of 400 ml. Her postoperative treatment followed the principle of enhanced recovery after the operation (ERAS). Her recovery was smooth without pancreatic leakage, biliary leakage, or liver failure. She was discharged on the 12th day postoperatively. The pathology revealed synchronous adenosquamous carcinoma of the gallbladder and common bile duct. This pathological result was finally given after a comprehensive discussion. The diagnosis of adenosquamous carcinoma was based on immunohistochemistry which showed both squamous cell carcinoma and adenocarcinoma. The diagnosis of dual original carcinoma instead of GBC with seeding metastasis to a common biliary duct was based on the fact of extensive lesion of the lower biliary duct without a clear boundary.

## Discussion

The indications for hepatopancreatoduonectomy (HPD) are locally advanced gallbladder cancer (GBC) and locally advanced extrahepatic cholangiocarcinoma (ECC) [3]. HPD is one of the most challenging procedures, which includes pancreaticoduodenectomy (PD) and hepatectomy. This combination results in a dramatic increase in the morbidity and mortality, especially in the risk of liver failure [4–6], which prevents its use. As reported, the morbidity of HPD is as high as 80%, and the in-hospital mortality may be more than 10% [3]. This rate is far higher than that of other operations, such as hepatectomy and PD. However, this increased mortality may be a result of the major hepatectomy, as the results from HPD with minor hepatectomy showed no increases in the mortality [10]. The survival results were promising for the patients who had HPD. The 3-year and 5-year overall survival rates were reported to be 48% and 37% [11], respectively, which were significantly higher than those of unresectable tumors [12]. The prolonged survival has encouraged surgeons to try this procedure.

As laparoscopic techniques have progressed, more and more complicated operations are completed by laparoscopy [13, 14] with an acceptable complication. Post-operation pancreatic leakage is considered to be the biggest obstacle to PD. Resent years, several modifications [15, 16] of pancreaticojejunostomy have been published to lessen the complication with success. Now, more and more laparoscopic pancreaticoduodenectomy (LPD) are reported with less pancreatic leakage and less blood loss [17]. The laparoscopic anatomic hepatectomy (LAH) also results in less blood loss and faster recovery [18]. In recent years, we have routinely carried out these complicated operations; therefore, we first adopted laparoscopy for HPD with success. LHPD has only been reported for 3 patients with locally advanced GBC or ECC, which were considered the main indications [19]. All the 3 operations were successful with acceptable complications which were listed in detail in Table 1. This was the first LHPD for concurrent GBC and ECC. Our experience showed that LHPD was safe and feasible. Our successful performance of the LHPD may be based on two factors. First, surgeon preparation should be emphasized. Surgeons who are interested in LHPD should be skilled in both LPD and LAH. Well-trained surgeons can perform LPD fluently with less morbidity [17]. LAH was also reported to lower morbidity following the intrahepatic Glissonean approach by skilled surgeons [18]. Only surgeons who were skilled at both of the complicated procedures can perform LHPD with safety. Second, the patient selection is important. As the increased morbidity may be secondary to the major hepatectomy, this combination of operations should be limited to a minor hepatectomy. Those patients who need a major hepatectomy should undergo a preoperative portal vein embolism (PVE). The results from patients who had a preoperative PVE showed less morbidity and less in-hospital mortality [20]. Preoperative bile drainage is controversial, as no significant advantage was shown according to published data [21]. Preoperative bile drainage should be recommended for those patients who need major hepatectomy or those with cholangitis [22], as jaundice may affect the remnant liver function [23]. Patients who plan to undergo LHPD should be at good performance status. The case reported here by our team was an elderly female patient who had no limitations in her daily life.

## Conclusions

According to the published cases and our experience, LHPD is safe and feasible by specially skilled surgeons. LHPD should be an option for selected patients with GBC or ECC.

## Data Availability

All data generated or analyzed during this study are included in this published article.

## Abbreviations

HPD: Hepatopancreaticoduodenectomy
LHPD: Laparoscopic hepatopancreaticoduodenectomy
GBC: Gallbladder cancer
ECC: Extrahepatic cholangiocarcinoma
ERAS: Enhanced recovery after an operation
PD: Pancreaticoduodenectomy
LPD: Laparoscopic pancreaticoduodenectomy
LAH: Laparoscopic anatomic hepatectomy
PVE: Portal vein embolism
